# Supplementary material for: Large ice loss variability at Nioghalvfjerdsfjorden Glacier, Northeast-Greenland
Source: Nat Commun. 2018 Jul 17;9:2768. doi: 10.1038/s41467-018-05180-x (PMC6050287; doi:10.1038/s41467-018-05180-x)
Supplement: Supplementary file 1 — Supplementary Information [file 41467_2018_5180_MOESM1_ESM.pdf]

Supplementary Information

Large ice loss variability and its causes at Nioghalvfjerdsfjorden Glacier, Northeast Greenland

by Mayer C. et al.

Supplementary Table 1: Measurements of along flow surface velocity and maximum tidal surface tilt (relative to neutral tide conditions) during the Midgardsormen experiment in 1998. The measurement period was 13 days.

Location	Velocity $m\ yr^{-1}$	max. tilt relative to NF1
NF1	619	1
NF0	384	0.11
NF8	238	0.03

Supplementary Table 2: The TanDEM-X raw DEMs used for this study. HoA – height of ambiguity of the bistatic InSAR acquisition. $<\sigma^0>$ the mean backscattering coefficient calculated over the floating ice tongue.

Date	Acquisition Item Id	Eff. Baseline [m]	HoA [m]	Relative orbit	Orbit direction	Incidence angle [deg]	$<\sigma^0>$ [dB]
2011/01/08	1010081_5	121.32	51.96	86	A	38.43	-6.8
2012/11/14	1107756_7	182.41	38.08	162	A	41.42	-7.6
2014/12/08	1249997_6	75.73	112.64	86	A	38.60	-5.3
2016/09/28	1381068_11	87.07	74.89	86	A	39.33	-12.1

Supplementary Table 3: The TanDEM-X – TanDEM-X elevation difference statistics: $SE_{\Delta z}$ is the estimated error remaining after the vertical co-registration of each raw TanDEM-X scene to the TanDEM-X global DEM reference; $SE_{\Delta h}$ is the statistical error of the difference measurement over the floating ice tongue; $\varepsilon_{\Delta h}$. is the overall uncertainty of the difference measurement; TCR is the buoyancy derived thickness change rate and ε_{TCR} denotes its error.

$Date_{slaveDEM}$ - $Date_{masterDEM}$	Mean [m]	Std. deviation [m]	No. of samples [pixels]	$SE_{\Delta h}$ [m]	$SE_{\Delta z}$ [m]	$\varepsilon_{\Delta h}$ [m]	TCR [$m\ yr^{-1}$]	ε_{TCR} [m]

2012/11/14-2011/01/08	-1.11	5.19	17309469	0.21	0.04	0.21	-5.99	1.12
2014/12/08-2012/11/14	-1.13	5.32	17310176	0.21	0.06	0.21	-5.46	1.04
2016/09/28-2014/12/08	-0.86	5.30	17979402	0.21	0.09	0.21	-4.77	1.17

Supplementary Table 4: Mean ice thickness changes and change rates across 79 North Glacier at the MGO cross profile, based on the comparison of airborne ground penetrating radar measurements (AGPR) in 1997 and TanDEM-X and ILATM (Operation Ice Bridge, 14 May 2012 and 29 April 2014) surface elevation measurements in 2012, 2014 and 2016. Errors are calculated according to the methods described above and the RMS-fit values provided in the ILATM data sets.

Period	AGPR - TDX	$\varepsilon_{\Delta h}$	Change rate	$\varepsilon_{\Delta h/t}$	AGPR - Icebridge ATM	$\varepsilon_{\Delta h}$	Change rate	$\varepsilon_{\Delta h/t}$
	[m]	[m]	[m yr^{-1}]	[m yr^{-1}]	[m]	[m]	[m yr^{-1}]	[m yr^{-1}]
1997-2012	86.7	1.27	5.78	0.09	73.4	4.57	4.89	0.31
1997-2014	89.5	1.27	5.27	0.08	88.0	4.57	5.18	0.27
1997-2016	98.1	1.27	5.16	0.07				

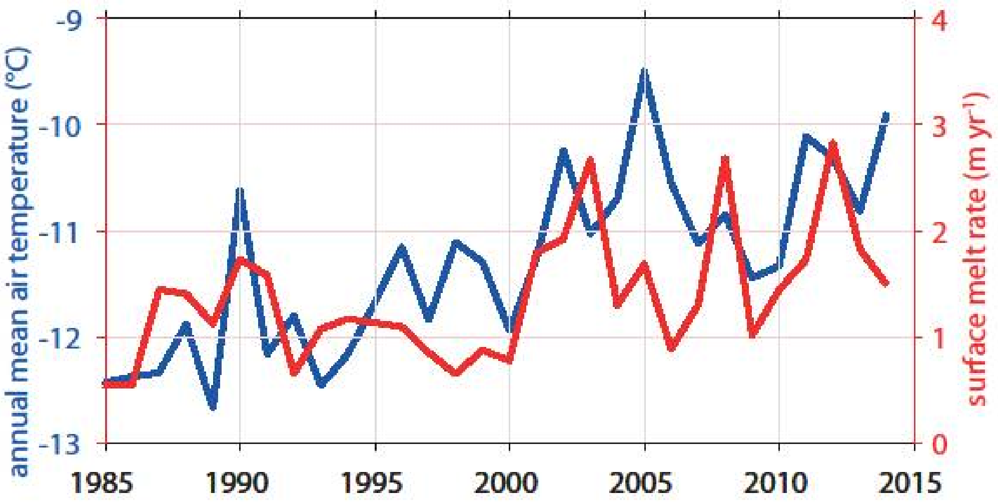

Supplementary Fig. 1: Time series of the annual mean air temperature from Danmarkshavn weather station (blue) and the computed annual surface melt rates for the floating part of 79 North Glacier (red).

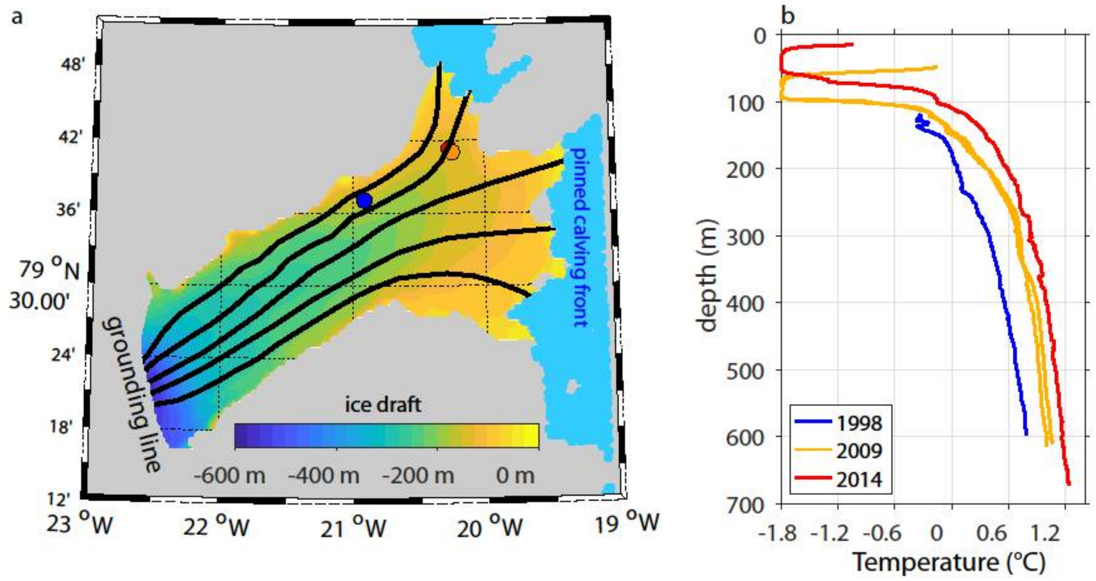

Supplementary Fig. 2: (a) Map of the ice draft of the 79 North Glacier based on RTopo-2 **(1)**. Grey shading indicates land, light blue shading indicates open ocean. Black lines mark the locations of the ice base profiles IB1, IB2, IB3, IB4, and IB5 used for the simulations with the ice-shelf plume model.

(b) Temperature profiles taken in the summer seasons in 1998, 2009, and 2014 through a glacial rift in the floating ice tongue (coloured circles in (a)).

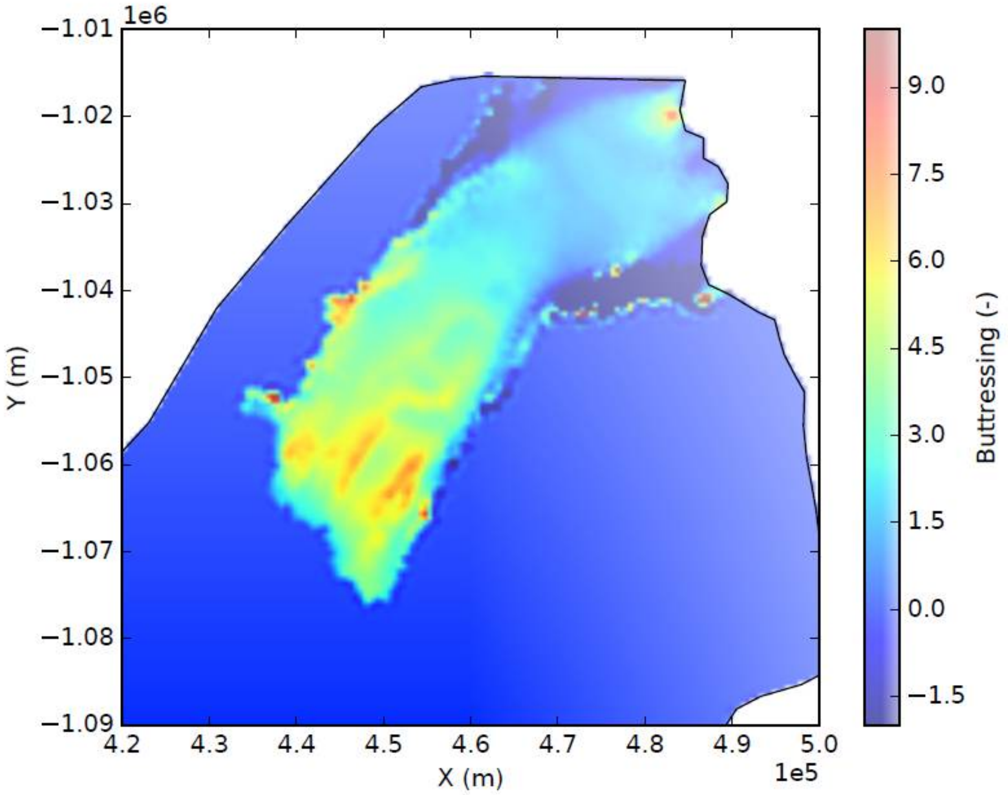

Supplementary Fig. 3: Flow buttressing for the floating part of 79 North Glacier and present day conditions. Values above 1 indicate strong buttressing, while negative values relate to pulling conditions.

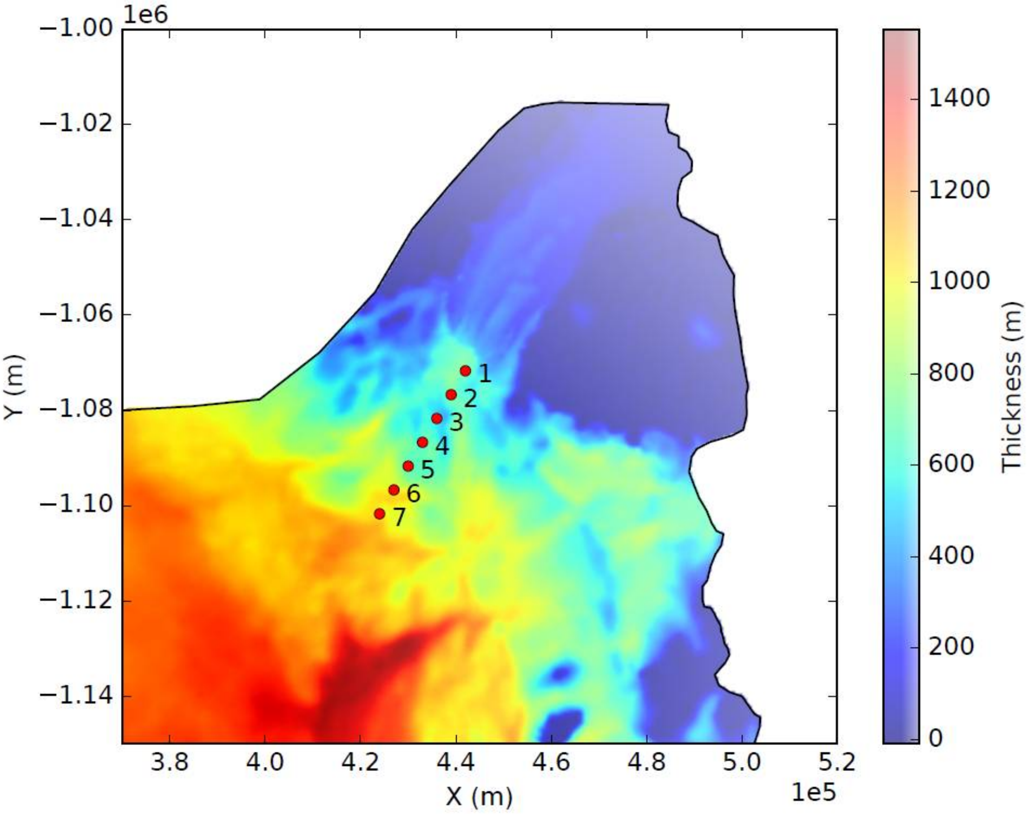

Supplementary Fig. 4: Ice thickness distribution for the model experiments of 79 North Glacier and the adjacent ice sheet. The sample points along a flow line are used to display changes for the scenario run (Supplementary Fig. 6).

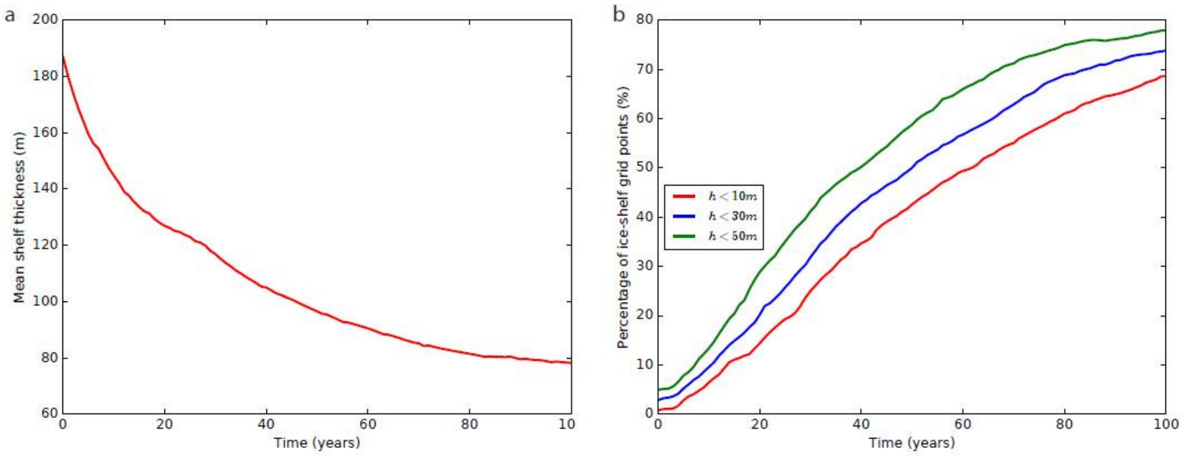

Supplementary Fig. 5: Mean ice thickness loss (left) and percentage of ice thicknesses for the floating part (right) during the 1.5 times standard mass balance run.

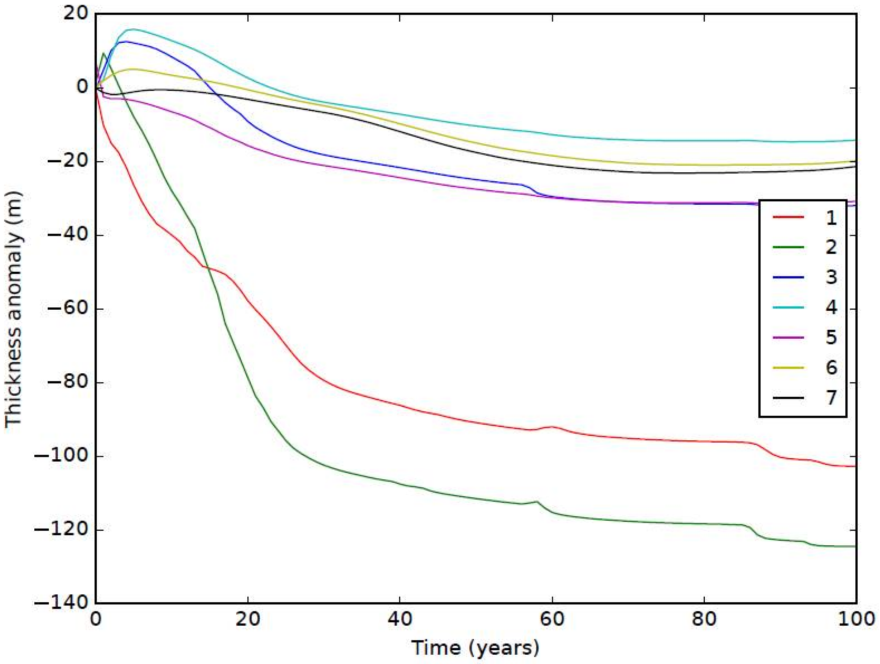

Supplementary Fig. 6: Ice thickness evolution along the flowline as depicted in Supplementary Fig. 4. A similar experiment for a total loss of the ice shelf results in ice thickness reductions of about 200 m within the first 10 km of the grounded flowline, while the ice loss increases to from 30 m to 50 m at 15-20 km upstream of the initial grounding line.

Supplementary References:

(1) Schaffer, J. et al. A global, high-resolution data set of ice sheet topography, cavity geometry, and ocean bathymetry. *Earth Syst. Sci. Data* **8,** 543-557. https://doi.org/10.5194/essd-8-543-2016 (2016).
